# Accuracy and reliability of self-administered visual acuity tests: Systematic review of pragmatic trials

**DOI:** 10.1371/journal.pone.0281847

**Published:** 2023-06-22

**Authors:** Arun James Thirunavukarasu, Refaat Hassan, Aaron Limonard, Shalom Vitreous Savant

**Affiliations:** 1 School of Clinical Medicine, University of Cambridge, Camybridge, United Kingdom; 2 Corpus Christi College, University of Cambridge, Cambridge, United Kingdom; 3 Sidney Sussex College, University of Cambridge, Cambridge, United Kingdom; 4 St John’s College, University of Cambridge, Cambridge, United Kingdom; University of Warmia, POLAND

## Abstract

**Background:**

Remote self-administered visual acuity (VA) tests have the potential to allow patients and non-specialists to assess vision without eye health professional input. Validation in pragmatic trials is necessary to demonstrate the accuracy and reliability of tests in relevant settings to justify deployment. Here, published pragmatic trials of these tests were synthesised to summarise the effectiveness of available options and appraise the quality of their supporting evidence.

**Methods:**

A systematic review was undertaken in accordance with a preregistered protocol (CRD42022385045). The Cochrane Library, Embase, MEDLINE, and Scopus were searched. Screening was conducted according to the following criteria: (1) English language; (2) primary research article; (3) visual acuity test conducted out of eye clinic; (4) no clinical administration of remote test; (5) accuracy or reliability of remote test analysed. There were no restrictions on trial participants. Quality assessment was conducted with QUADAS-2.

**Results:**

Of 1227 identified reports, 10 studies were ultimately included. One study was at high risk of bias and two studies exhibited concerning features of bias; all studies were applicable. Three trials—of DigiVis, iSight Professional, and Peek Acuity—from two studies suggested that accuracy of the remote tests is comparable to clinical assessment. All other trials exhibited inferior accuracy, including conflicting results from a pooled study of iSight Professional and Peek Acuity. Two studies evaluated test-retest agreement—one trial provided evidence that DigiVis is as reliable as clinical assessment. The three most accurate tests required access to digital devices. Reporting was inconsistent and often incomplete, particularly with regards to describing methods and conducting statistical analysis.

**Conclusions:**

Remote self-administered VA tests appear promising, but further pragmatic trials are indicated to justify deployment in carefully defined contexts to facilitate patient or non-specialist led assessment. Deployment could augment teleophthalmology, non-specialist eye assessment, pre-consultation triage, and autonomous long-term monitoring of vision.

## Introduction

Visual acuity (VA) is a measure of the functional resolution of vision, and is assessed before every ophthalmological, optometric, and orthoptic examination to inform decision making. Generally, distance VA assessment involves a clinician appraising the smallest optotype the patient can read while at a standard distance away from an illuminated chart. VA is reported in one of three forms: Snellen fraction, where the numerator denotes the distance between participant and chart and denominator denotes the distance at which ‘ideal’ sight can distinguish the smallest letter identified by the patient (6/6 or 20/20 being ideal, higher denominators corresponding to worse vision); logarithm of the minimum angle of resolution (logMAR) expressed as a real number (0 logMAR being ideal, higher numbers corresponding to worse vision); or letters read, a positive integer where 1 letter is the equivalent of 0.02 logMAR progression (85 letters being ideal, lower numbers corresponding to worse vision). The latter two measures are generated by using the Early Treatment for Diabetic Retinopathy Study (ETDRS) chart, whereas the former measure is generated when using the older Snellen chart. Below, VA is referred to in terms of logMAR throughout.

Self-administered VA tests provide patients with a means of monitoring their vision without having to be examined by an eye health professional. These tests may augment telehealth services, as VA assessment is an integral part of any eye examination. Adoption of self-administered VA tests may reduce the burden on strained ophthalmology resources by enabling non-specialists to triage with knowledge of visual function; by improving referral quality with provision of VA data; and by facilitating autonomous monitoring of vision by patients with chronic eye conditions (who otherwise require frequent clinic appointments) [1–3].

Many remote visual acuity tests have been developed, but most have been validated with administration in real time by a trained clinician, as required with conventional VA assessment with Snellen or ETDRS chart [4–6]. As the requirement for clinical examination limits the usefulness of ophthalmic telehealth services, platforms facilitating further examination without physical attendance will serve as important components of any improved suite for remote consultation [3,6]. Pragmatic trials are essential to demonstrate that remote tests are useful for generating actionable VA data without skilled supervision—artificial environments are expected to inflate accuracy and reliability [7,8]. Validation data generated in unrealistic settings provides weaker justification for subsequent clinical deployment than results generated in real-world conditions [8]. The aim of pragmatic trials is to gauge effectiveness—performance in real world conditions—rather than efficacy, or performance in an ideal environment.

Here, a systematic review was undertaken to identify pragmatic trials of remote self-administered VA tests; appraise the quality of their validation data; and compare these tests to conventional visual acuity testing. Specifically, the accuracy and reliability of VA self-tests were gauged to help establish the clinical utility of available platforms. All trials were pragmatic in that remote tests were administered without real-time clinical input, away from idealised but artificial conditions. This evidence synthesis serves as a point of reference for clinicians, patients, and policy makers interested in identifying appropriate platforms to facilitate visual acuity assessment without requiring eye health service involvement.

## Materials and methods

### Search and screening

This systematic review adhered to PRISMA guidance, according to a prospectively registered protocol on PROPERO (identifier CRD42022385045). On 23 December 2022, The Cochrane Library, Embase (via OVID), MEDLINE (via PubMed), and Scopus were searched for the following: ("visual acuity") AND ("remot*" OR "portable" OR "home based") AND ("test" OR "assessment" OR "examination"). Previously published reviews were also searched for relevant studies [4–6,9]. Duplicates were removed by a single researcher using Zotero (version 6.0.19-beta.15+6374aea1c; Digital Scholar, Vienna, Virginia, USA). Abstract and full text screening were undertaken by two independent researchers in Rayyan, with a third researcher acting as arbiter to resolve disagreement [10]. The following inclusion criteria were employed, with no restrictions on participant characteristics or test modality:

1. Record is written in the English language
2. Record is a peer-reviewed primary research article
3. Study examines a visual acuity test undertaken out of eye clinic (*i*.*e*. remotely).
4. Remote test does not require a clinically trained administrator (*i*.*e*. patient-led).
5. Remote patient-led test is compared to clinical or repeated remote visual acuity measurements to assess accuracy or reliability, respectively.

### Data extraction and analysis

Risk of bias and concerns regarding applicability were appraised with the QUADAS-2 framework by a single researcher, with a second researcher verifying each appraisal [11]. One researcher undertook data extraction for each included study, with a second independent researcher verifying every entry. Data gathered included details about participants, index tests, reference tests, measured outcomes, and study designs; and for index test-retest reliability and accuracy (*i*.*e*. comparison to clinical reference test), the bias and limits of agreement of Bland-Altman plots, intraclass correlation coefficients (ICCs) and *p* value, and *t*-test *p* value. ICCs were only reported for test-retest agreement, as they are a poor method for comparing different tests [12,13]. For consistency, bias was expressed as the mean difference between reference and index test, such that positive values indicated that the reference test tended to provide a higher value (*i*.*e*. where the index test overestimated visual acuity). Where studies provided individual participants’ VA data without further analysis, the two-way random effects intraclass correlation coefficient (ICC) was calculated, and unpaired two-samples *t*-test was conducted. For studies exhibiting Bland-Altman plots without reporting figures for the bias and limits of agreement, manual interpolation was conducted with WebPlotDigitizer (version 4.6.0; Ankit Rohatgi, Pacifica, California, USA). Meta-analysis was planned but ultimately precluded by a lack of trials testing the same platform. Data extraction and quality assessment were conducted in in Microsoft Excel for Mac (version 16.57; Microsoft Corporation, Redmond, Washington, USA). Data analysis was conducted in R (version 4.1.2; R Foundation for Statistical Computing, Vienna, Austria) [14–16]. Tables were produced in Microsoft Excel for Mac. Figures were produced in R and modified with Affinity Designer (version 1.10.4; Pantone LLC, Carlstadt, New Jersey, USA).

## Results

**Fig 1:**
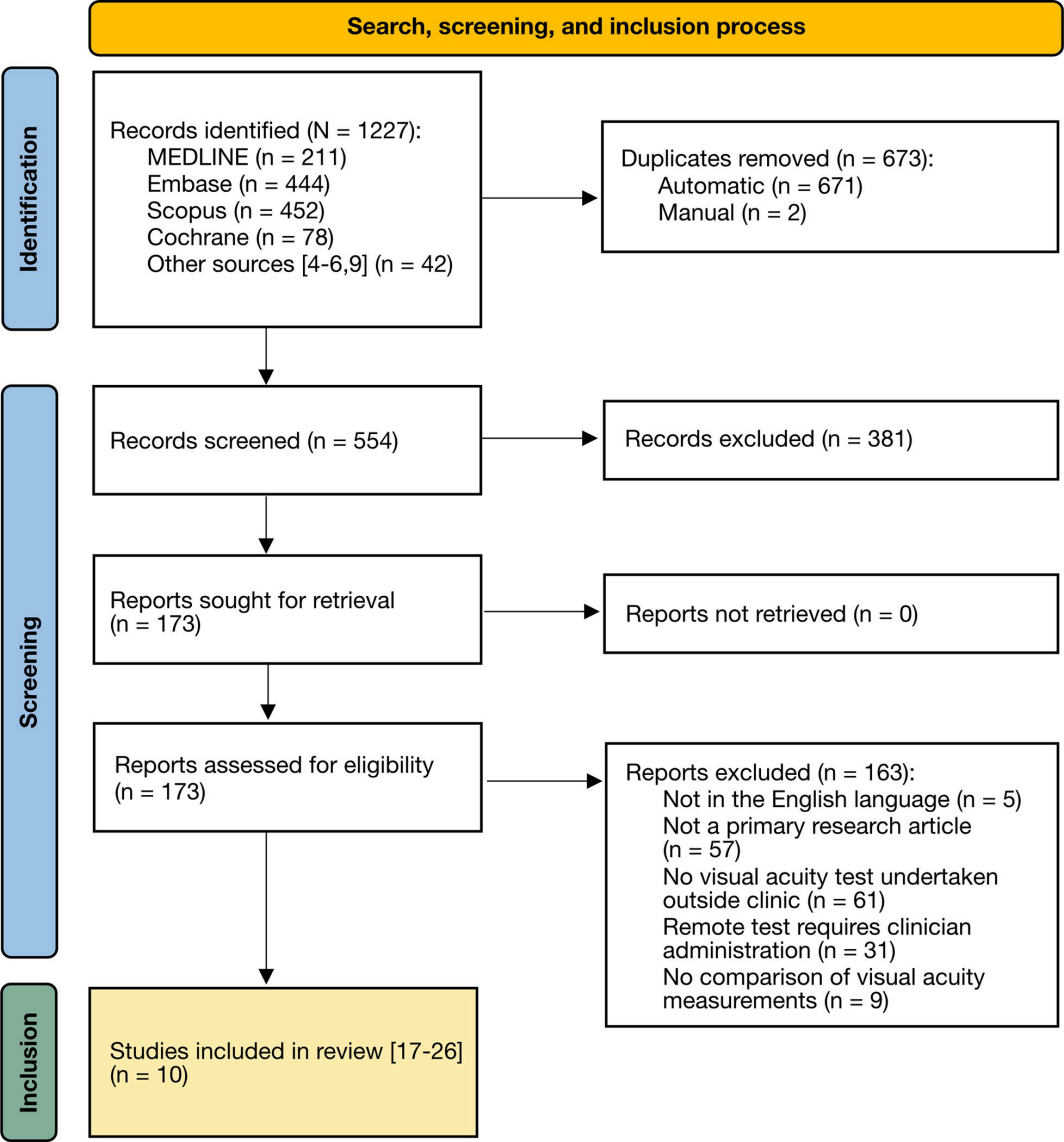
PRISMA flowchart. Illustrating the literature search, screening process, and articles included in this review. PRISMA = Preferred Reporting Items for Systematic Reviews and Meta Analyses; MEDLINE = Medical Literature Analysis and Retrieval System Online.

The undertaken literature search and screening process is summarised in Fig 1. Ten studies were included from 1227 identified reports [17–26]. Fulfilling criterion (3) necessitated that trials were pragmatic in that remote tests were conducted out of the eye clinic [27]. Hyperacuity tests and survey-based self-assessment were excluded [28–31]. To fulfil criterion (4), tests had to be patient-led: while tests administered by parents for paediatric patients were acceptable, involvement of clinicians or other trained personnel justified exclusion [32–34]. Criterion 5 mandated exclusion of studies involving tests which did not provide visual acuity measurements which could be compared to conventional clinical assessment or repeated remote measurement [35–37].

**Fig 2:**
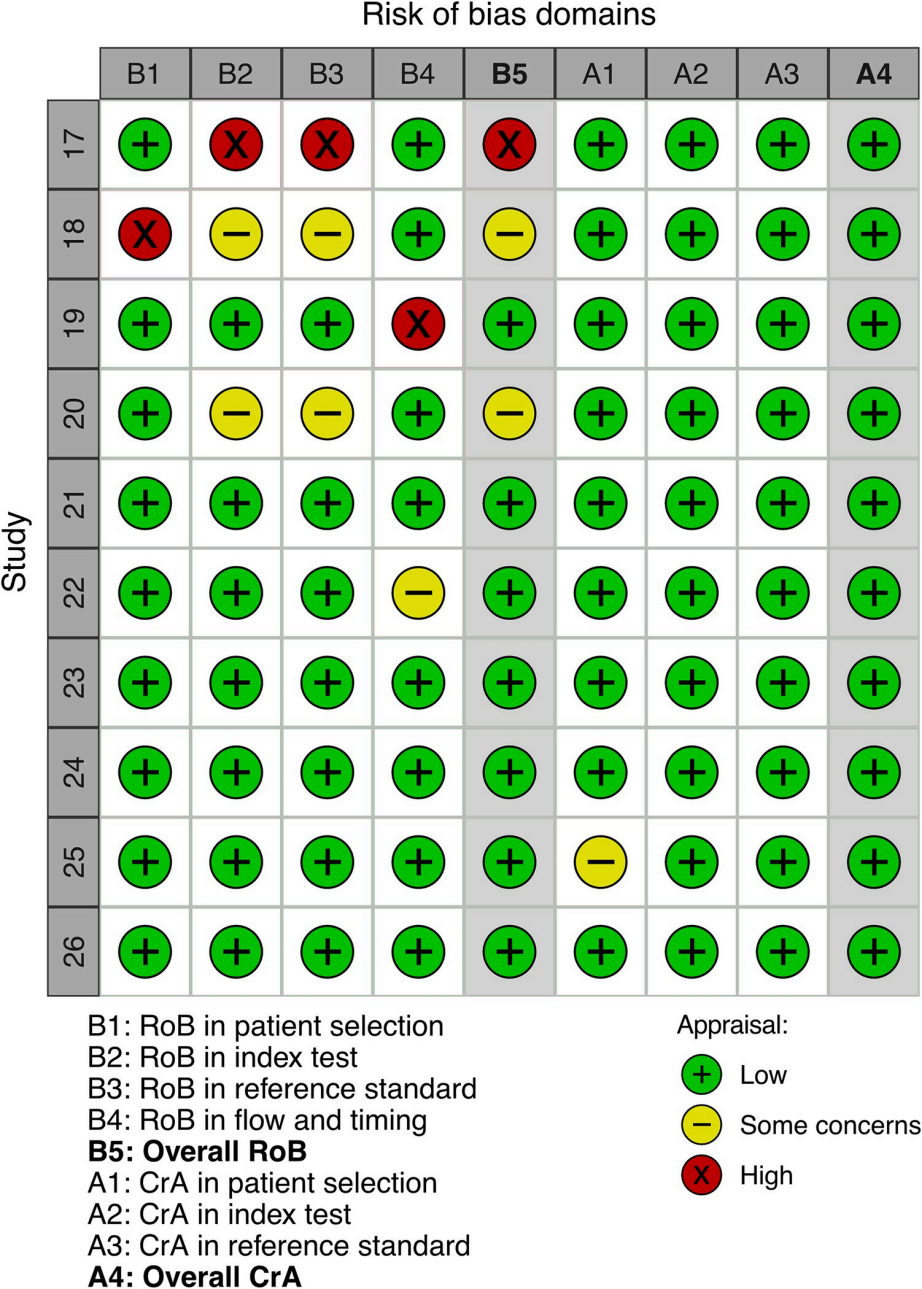
Risk of bias and inapplicability appraisals for each included study. Appraised with the QUADAS-2 framework. QUADAS-2 = Quality Assessment of Diagnostic Accuracy Studies 2; RoB = risk of bias; CrA = concerns regarding applicability.

Study characteristics are summarised in Table 1. Most studies were prospective cross-sectional surveys, with just one retrospective case-control study. 6 of 10 studies reported conflicts of interest, suggesting that many validation studies were not undertaken by research teams independent from the trialled product—a potential source of reporting bias. However, none of the included studies received private funding, such as from product manufacturers. The number of participants ranged from 7 to 148 (median = 50.5). Reported participant age ranged from 3 to 95 years old—spanning most of the paediatric and adult ophthalmology case load. Most trialled tests required access to digital devices: exceptions required a paper chart or custom-built e-device; both provided by the investigators [19,26]. One study required patients to print a physical chart sent to their digital device [24]. Risk of bias judged with QUADAS-2 was generally low, as illustrated in Figs 2 and S1. No major concerns regarding applicability were highlighted during QUADAS-2 appraisal, likely due to stringent inclusion criteria ensuring all studies applied patient-led tests remotely.

**Table 1:** Characteristics of each of the included studies.

Citation	Funding	Conflict of interest	Country of corresponding author	Participants	Index test	Index test hardware	Reference test	Outcome	Study design
Adyanthaya and B, 2022 [17]	None	None	India	N = 148; 6–14 years old; all had non-acute ocular symptoms	(1) iSight Professional (2) Peek Acuity	Apple and Android smartphones	Snellen chart at 6m in clinic	Distance BCVA in logMAR	Cross-sectional survey
Almagati and Kran, 2021 [18]	Public	Previous co-authorship between reviewer and co-author	United States of America	N = 7; 3–7 years old; low vision clinic patients: 3 with cerebral visual impairment, 4 without	FrACT Landolt-C	Digital internet connected devices	Most recent clinical assessment	Distance BCVA in logMAR	Case control
Bellsmith et al, 2022 [19]	Public	None	United States of America	N = 121; 18–78 years old; eye clinic patients with VA of 20/200 Snellen or better	(1) University of Arizona/Banner Eye Health Chart (2) Verana Vision Test (3) Farsight.care	Apple device, internet-connected computer, or paper chart	Electronic Snellen chart in clinic	Distance BCVA in logMAR	Cross-sectional survey
Chen et al, 2022 [20]	None	Co-authors invented and hold patent for the trialled test; co-author consults for KYS Vision	United States of America	N = 25; all over 18 years old; retina clinic patients with VA of 20/200 Snellen or better	Acustat	Digital internet connected devices	Snellen chart in clinic	Near BCVA in logMAR	Cross-sectional survey
Chen et al, 2021 [21]	Public	Co-author paid by Zeiss, Allergan, Vanda, and Long Bridge Medical	United States of America	N = 45; glaucoma clinic patients with VA better than 20/125	Letter Distance Chart PDF document	Digital device	Electronic Snellen chart in clinic	Distance BCVA in logMAR	Cross-sectional survey
Painter et al, 2021 [22]	Not stated	None	United Kingdom	N = 15; paediatric ophthalmology patients with a previously recorded VA	(1) iSight Professional (2) Peek Acuity	Smartphone or tablet	Most recent clinical assessment	Distance BCVA in logMAR	Cross-sectional survey
Pathipati et al, 2016[23]	None	None	United States of America	N = 27; emergency department patients with an ophthalmology consult ordered	Paxos Checkup	Fourth generation Apple iPod Touch	(1) Rosenbaum near card (2) Snellen chart at 20 feet in ED	Near and Distance BCVA in logMAR	Cross-sectional survey
Siktberg et al, 2021 [24]	Public	Co-author paid by Alcon	United States of America	N = 108; 18–85 years old; patients with ophthalmology appointment scheduled with no prior recorded VA worse than 20/200	ETDRS vision chart PDF document	Internet-connected device and a printer	ETDRS chart at 4m in clinic	Distance BCVA in ETDRS letters	Cross-sectional survey
Thirunavukarasu et al, 2022 [25]	Public	Co-author invented and applied for patent for the trialled test; co-author is founding director of Cambridge Medical Innovation Ltd	United Kingdom	N = 120; 5–87 years old; patients with ophthalmology appointment scheduled with VA better than 0.8 logMAR	DigiVis	Digital internet connected devices	Conventional assessment in clinic	Distance BCVA in logMAR	Cross-sectional survey
Van Der Star et al, 2022 [26]	None	Co-author consults for DORC International, Dutch Ophthalmic USA, and SurgiCube International; patent for trialled test pending	United States of America	N = 56; 16–95 years old; patients with previous intraocular surgery or chronic ocular disease	Custom-built e-device	Custom built e-device with miniaturised Snellen chart virtually projected at 20 ft	Snellen chart at 20 feet in clinic	Distance BCVA in Snellen fraction	Cross-sectional survey

BCVA = best corrected visual acuity; VA = visual acuity; logMAR = logarithm of the minimum angle of resolution; PDF = portable document format; ETDRS = Early Treatment of Diabetic Retinopathy Study.

All studies gauged accuracy by comparing remote measurements to assessment in clinic (Table 2). The reference test was not consistently defined in three studies [18,22,25], and Snellen chart was used in four studies [19–21,23,26]; as opposed to the gold-standard Early Treatment for Diabetic Retinopathy Study (ETDRS) chart which was used consistently in just one study [24]. One study trialling FrACT provided individualised data which enabled calculation of the bias and intraclass correlation coefficient, but its small sample size and retrospective design were discussed by the authors as significant limitations necessitating further validation; and statistics were not calculated by the authors themselves as their clinical measurements were not recent enough to serve as a fair control [18]. One trial of a custom e-device did not report any statistical analysis or individual data [26].

**Table 2:** Accuracy data.

Citation	Index test	Reference test	N	Bias (95% CI)	LLOA (95% CI)	ULOA (95% CI)	*t*-test *p* value
Adyanthaya and B, 2022 [17]	iSight Professional	Snellen chart	286 eyes of 148 patients	0.06 (0.04 to 0.08)	0.04	0.1	
Adyanthaya and B, 2022 [17]	Peek Acuity	Snellen chart	286 eyes of 148 patients	0.07 (0.05 to 0.09)	0.04	0.1	
Almagati and Kran, 2021 [18]	FrACT	Clinic assessment	14 eyes (binocular assessment) of 7 patients	-0.09			0.63
Bellsmith et al, 2022 [19]	University of Arizona/Banner Eye Health Chart	Snellen chart	137 eyes of <121 patients	-0.07 (-0.1 to -0.04)	-0.39 (-0.44 to -0.34)	0.25 (0.20 to 0.30)	
Bellsmith et al, 2022 [19]	Verana Vision Test	Snellen chart	147 eyes of <121 patients	-0.12 (-0.15 to -0.09)	-0.50 (-0.55 to -0.44)	0.26 (0.21 to 0.32)	
Bellsmith et al, 2022 [19]	Farsight.care	Snellen chart	146 eyes of <121 patients	-0.13 (-0.16 to -0.10)	-0.53 (-0.58 to -0.46)	0.27 (0.21 to 0.33)	
Chen et al, 2022 [20]	Acustat	Snellen chart	50 eyes of 25 patients		-0.2278	0.2235	0.8997
Chen et al, 2021 [21]	Letter Distance Chart PDF document	Snellen chart	45 eyes of 45 patients	-0.02	-0.31	0.26	0.28
Chen et al, 2021 [21]	Letter Distance Chart PDF document	Snellen chart	42 eyes of 42 patients	-0.02	-0.31	0.27	0.32
Painter et al, 2021 [22]	iSight Professional or Peek Acuity	Clinic assessment	30 eyes of 15 patients	-0.14	-0.88	0.6	
Pathipati et al, 2016 [23]	Paxos Checkup	Rosenbaum near card	51 eyes from 27 patients	-0.06			0.264
Siktberg et al, 2021 [24]	ETDRS vision chart PDF document	ETDRS chart	209 eyes from 108 patients	0.078			
Thirunavukarasu et al, 2022 [25]	DigiVis	Clinic assessment	120 eyes from 120 patients	-0.001 (-0.017 to 0.015)	-0.175 (-0.202 to -0.147)	0.173 (0.146 to 0.201)	
Van Der Star et al, 2022 [26]	Custom-built e-device	Snellen chart	72 eyes from 56 patients				

Comparing remote index tests to clinical reference tests. CI = confidence interval; LLOA = lower 95% limit of agreement; ULOA = upper 95% limit of agreement; ICC = intraclass correlation coefficient; PDF = portable document format; ETDRS = Early Treatment of Diabetic Retinopathy Study.

**Fig 3:**
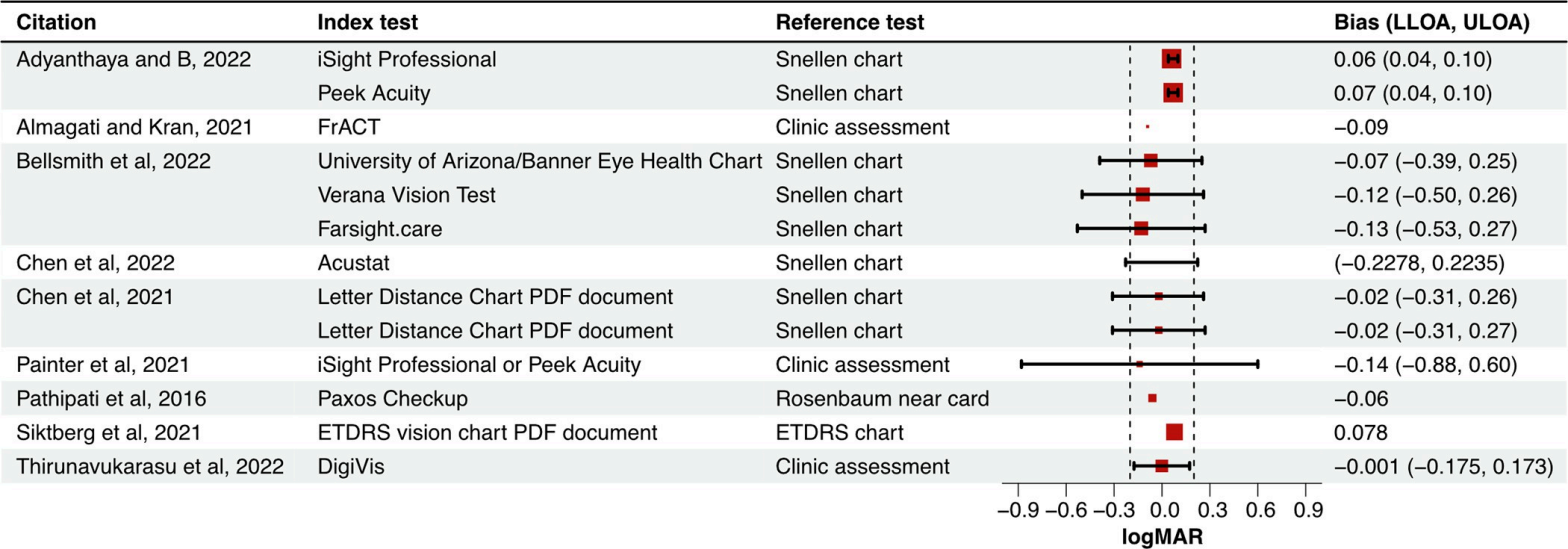
Forest plot summarising Bland-Altman analyses of accuracy. LLOA = lower 95% limit of agreement; ULOA = upper 95% limit of agreement; PDF = portable document format; ETDRS = Early Treatment of Diabetic Retinopathy Study; logMAR = logarithm of the minimum angle of resolution.

Eight studies provided Bland-Altman statistics, corresponding to trials of twelve remote VA tests (Fig 3) [17,19–25]. Of these, six studies (ten trials) provided 95% lower and upper limits of agreement (LLOA and ULOA respectively) [17,19–22,25]. LOA of Isight pro, Peek Acuity, and DigiVis lay within ±0.2 logMAR in three trials [17,25]. The remaining seven trials corresponded to University of Arizona/Banner Eye Health Chart, Verna Vision Test, Farsight.care, Acustat, Letter Distance Chart PDF document (twice), and Isight pro or Peek Acuity pooled together [19–22]. One study did not report the bias; of the remaining nine studies, three (containing six trials) provided 95% confidence intervals [17,19,25]. Isight pro and Peek Acuity exhibited significantly higher bias than 0 logMAR (index test estimated worse acuity) [17]; University of Arizona/Banner Eye Health Chart, Verana Vision Test, and Farsight.care exhibited significantly lower bias than 0 logMAR (index test estimated better acuity) [19]; and DigiVis exhibited no statistically significant bias [25]. Two studies (4 trials) reported correlation coefficients, but these cannot be used to appraise agreement between different tests [12]. Four studies’ (five trials) *t*-tests comparing measurement methods all reported *p*-values above 0.25 [18,20–23].

Two trials reported test-retest reliability: one trialling DigiVis [25], and one trialling Isight pro and Peek Acuity in a pooled analysis [22]. The former reported Bland-Altman statistics and ICC, whereas the latter only reported the coefficient of repeatability (CoR) (Table 3). DigiVis exhibited a bias equivalent to 0, LOA of ±0.12 logMAR (6 letters), and ICC of 0.922 [25]. In a pooled analysis, Isight pro and Peek Acuity exhibited a CoR of 0.03 logMAR [22].

**Table 3:** Test-retest agreement.

Citation	Test	N	Bias (95% CI)	LLOA (95% CI)	ULOA (95% CI)	CoR (95% CI)	ICC (95% CI)	ICC *p* value
Painter et al, 2021 [22]	iSight Professional or Peek Acuity	26 eyes of 13 patients				0.03 (-0.08 to 0.04)		
Thirunavukarasu et al, 2022 [25]	DigiVis	105 eyes from 105 patients	0.001 (-0.011 to 0.013)	-0.121 (-0.142 to -0.101)	0.124 (0.103 to 0.144)		0.922 (0.887 to 0.946)	<0.001

Assessing the reliability of remote tests. CI = confidence interval; LLOA = lower 95% limit of agreement; ULOA = upper 95% limit of agreement; CoR = coefficient of repeatability; ICC = intraclass correlation coefficient.

## Discussion

To justify adoption of remote self-administered VA tests, there must be convincing evidence that the proposed platform meets regulatory safety standards, is effective enough to fulfil its clinical function, is accessible to patients—with appropriate mechanisms to serve those unable to use the platform, and is economically viable [38]. Facilities for VA self-assessment may be useful in a number of domains: improving the capacity and capability of teleophthalmology clinics, empowering patients with the ability to monitor their own vision rather than attend regular appointments; enabling non-eye specialists to obtain useful information for a referral to ophthalmology; and giving eye units a tool to facilitate pre-attendance triage of eye casualty cases [2,3]. In all cases, it is essential that tests are accurate and reliable, exhibiting agreement with clinical assessment and with repeated remote measurement, respectively.

In ideal conditions, chart-based VA still exhibits considerable variation, with 95% LOA approaching 0.09 logMAR; and in clinical settings LOA broaden to at least ±0.15 logMAR [7,39]. Clinical variation is greater as different examinations may be more or less demanding of patient effort, and may or may not test to majority failure (*i*.*e*. ≥3 errors on 1 line) [40]. Where both index and reference test exhibit variation, the utility of analyses restricted to *t*-tests or correlation coefficients is limited. Bland-Altman analysis compensates for bivariate variation by quantifying 95% LOA, which provides metrics of measurement dispersal which can be compared to gold-standard tests. Studies failing to conduct appropriate analyses fail to provide evidence of validation—it is not possible to ascertain whether observed variation is clinically acceptable or not. Acceptable 95% LOA should compare well with those exhibited by conventional clinical chart-based tests: below ±0.2 logMAR and ideally approaching ±0.15 logMAR [39,40]. Bias should be close to zero—statistically significant deviation (*e*.*g*. if confidence intervals do not cross zero) indicates a systematic error. High correlation is expected—over 0.7 in terms of Pearson’s or intraclass correlation coefficients [39,41].

Here, DigiVis was the only test exhibiting undisputed 95% LOA within 0.2 logMAR, no significant bias, and high correlation between remotely and clinically assessed VA [25]. iSight Professional and Peek Acuity exhibited 95% LOA within 0.2 logMAR in one of two studies, but this study was judged to be at a high risk of bias [17]. However, in the trial finding greater LOA, pooling of results from both tests may have affected calculated accuracy [22]. Just two studies reported test-retest agreement. One study indicated that DigiVis measurements are very reliable [25]; while another indicated good agreement between repeated iSight Professional and Peek Acuity measurements, albeit with fewer statistics provided [22]. Again, pooling of iSight Professional and Peek Acuity data may have affected the result.

All three tests with positive validation data had no requirement for real-time administration by a trained clinician. Therefore, all three may be used to improve the capability of telehealth services and eye assessment by non-specialists such as general practitioners and emergency department clinicians. However, as some patients in the DigiVis trial conducted the remote test in clinical settings, it is difficult to conclude with certainty that deployment for home-based assessment is justified [25]. All three tests relied on digital devices, accessible by most of the world’s population [42]. However, as uptake of smartphone-based vision tests correlates negatively with older age and worse vision, healthcare providers should be mindful of patients’ capacity to access and complete remote VA assessment to ensure their care and outcomes are not adversely affected [37].

This review was limited by three factors: **(1)** Inconsistent and incomplete statistical analysis made establishing the accuracy and reliability of trialled VA tests challenging. Deduction of the direction of bias was often based on limited prose descriptions—this is a potential source of error but would not affect conclusions significantly as bias was always close to zero. **(2)** Descriptions of the setting of the remote index test was often unclear, making the full-text screening process more difficult. Included studies all mentioned a test undertaken outside the eye clinic and did not state that all tests were conducted in clinical or ideal settings. **(3)** Most studies did not use Bailey-Lovie or ETDRS charts which are accepted as more accurate and precise for clinical research. While this may inflate variability in the reference test and consequently inflate calculated accuracy of the remote index tests, use of Snellen chart may not be a specific weakness as it remains widespread in clinics around the world [43,44].

Although promising technology has been developed to remotely assess VA, very few studies have demonstrated that patient-led assessment outside the eye clinic is feasible. DigiVis, iSight Professional, and Peek Acuity all have validation data demonstrating equivalence with clinical assessment, with the former being best justified due to conflicting results regarding the latter two tests. Further pragmatic trials are required to demonstrate the accuracy and reliability of remote VA assessment to justify deployment at scale, ideally using gold standard clinical assessments to maximise the validity of conclusions—ongoing trials and more recent reports may fill this gap in the literature base [45–47]. However, as these trials are often organised by test manufacturers, owners, or patent-holders, independent researchers may seek to run their own studies to ensure validation data are unbiased. Further work is also required to establish the precise populations in which tests exhibit acceptable accuracy and reliability, as this may vary over range of vision, disease state, and age. Finally, work is indicated to explore the feasible use-cases of remote VA tests: in-person examination remains essential for a comprehensive ophthalmological assessment, but remote VA tests may nevertheless improve service provision and reduce the strain on limited clinic resources—particularly if incorporated alongside other emerging digital health tools [48]. Validated self-administered VA tests have the potential to augment teleophthalmology services, pre-consultation triage, long-term monitoring, as well as non-specialist assessment and reporting of eye problems [3].

## Supporting information

S1 Checklist PRISMA 2009 checklist. (PDF)

S1 Fig Summarised risk of bias and inapplicability. Appraised with the QUADAS-2 framework. QUADAS-2 = Quality Assessment of Diagnostic Accuracy Studies 2; RoB = risk of bias; CrA = concerns regarding applicability.
(TIF)
