# Supplementary figures and images for: Accuracy and reliability of self-administered visual acuity tests: Systematic review of pragmatic trials
Source: PLoS One. 2023 Jun 22;18(6):e0281847. doi: 10.1371/journal.pone.0281847 (PMC10286971; doi:10.1371/journal.pone.0281847)

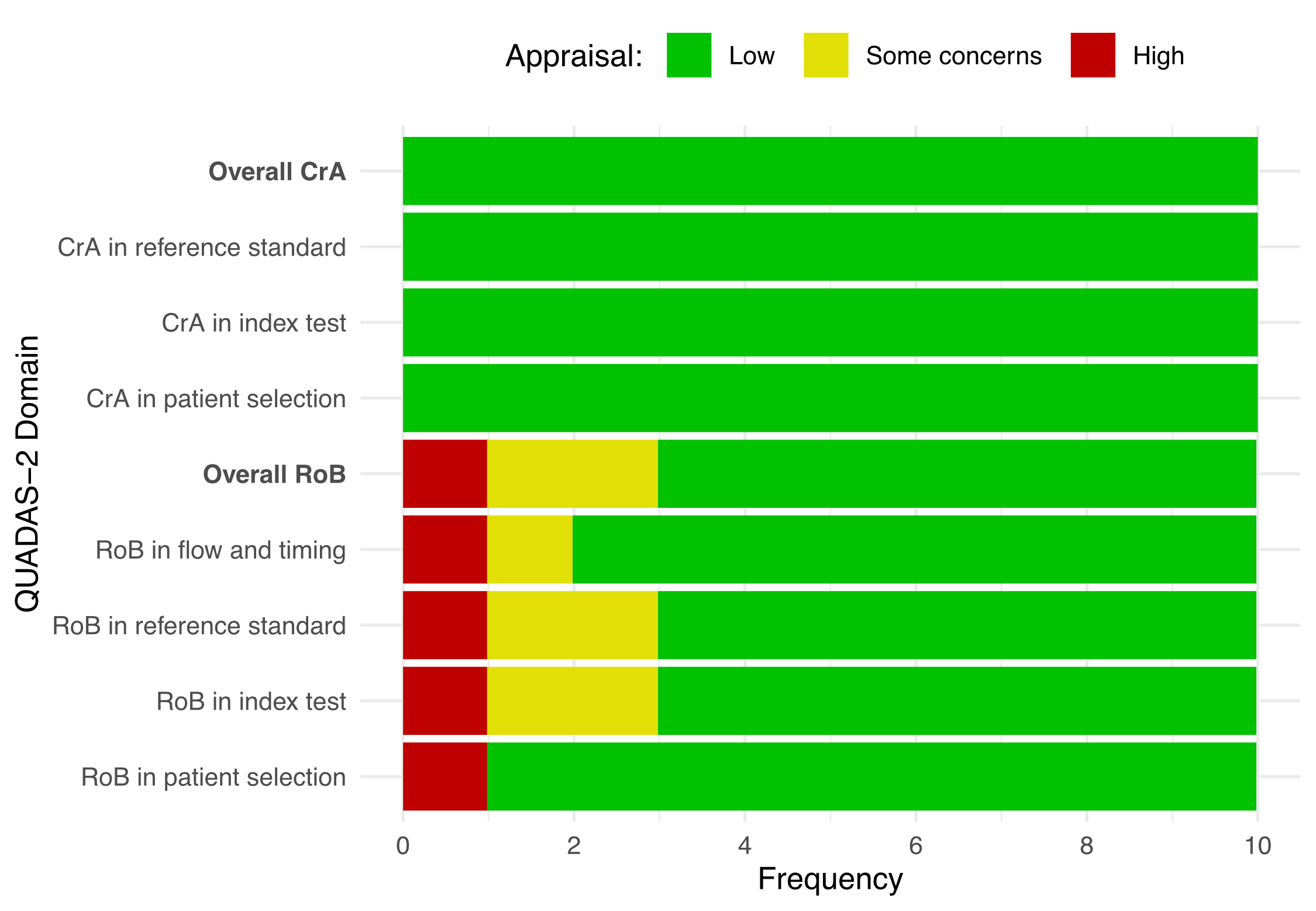

Supplement: S1 Fig — Appraised with the QUADAS-2 framework. QUADAS-2 = Quality Assessment of Diagnostic Accuracy Studies 2; RoB = risk of bias; CrA = concerns regarding applicability. (TIF) [file pone.0281847.s002.tif]
